# Axillary Reverse Mapping Improves Quality of Life by Significantly Reducing Clinically Relevant Lymphedema After Axillary Lymph Node Dissection in Older Women with Breast Cancer

**DOI:** 10.3390/curroncol33040212

**Published:** 2026-04-10

**Authors:** Merve Tokocin, Turan Pehlivan, Atilla Celik

**Affiliations:** Department of General Surgery, University of Health Sciences Türkiye, Istanbul Bacılar Training and Research Hospital, Istanbul 34100, Turkey; drturanpehlivan@hotmail.com (T.P.); dratillacelik@yahoo.com (A.C.)

**Keywords:** axillary reverse mapping, breast cancer-related lymphedema, quality of life, older adults, ALND, lymphedema prevention, indocyanine green, oncologic safety

## Abstract

**Background:** Breast cancer-related lymphedema (BCRL) is one of the most debilitating long-term morbidities after axillary lymph node dissection (ALND), severely impairing quality of life through reduced mobility, independence, and chronic burden, especially in older women. Axillary reverse mapping (ARM) aims to preserve upper extremity lymphatics while maintaining oncologic safety. Evidence in older adult populations with long-term follow-up remains limited. **Methods:** This retrospective cohort study included 138 female patients (median age 72.5 years) undergoing ALND for invasive breast cancer between January 2018 and January 2024. Patients were divided into ARM (n = 72) and non-ARM (n = 66) groups. BCRL was graded 0–3 according to adapted International Society of Lymphology (ISL) criteria (2013 consensus document). Assessments were performed preoperatively and at 3, 6, 12, 24, 36, 48, and 60 months using blinded circumference measurements and bioimpedance spectroscopy. **Results:** Baseline characteristics were comparable. Mean follow-up was 46.5 ± 8.8 months. Clinically relevant BCRL (Grades 2–3) was dramatically lower in the ARM group (18.1% vs. 60.6%, *p* < 0.0001), while subclinical changes (Grade 1) were similar (31.9% vs. 27.3%, *p* = 0.55). Kaplan–Meier analysis showed significantly better clinically relevant lymphedema-free survival with ARM (log-rank *p* = 0.00019), with curve separation after 30–40 months—indicating a sustained long-term benefit for quality of life in this frail population. Recurrence rates were comparable (8.3% vs. 10.6%, *p* = 0.776). Multivariable Cox regression confirmed ARM as an independent protective factor (adjusted HR 0.22, 95% CI 0.11–0.44, *p* < 0.0001). **Conclusions:** In older women with breast cancer, ARM significantly reduces clinically relevant lymphedema—a major determinant of long-term quality of life—without compromising oncologic safety. These findings support the routine consideration of ARM during ALND to preserve upper-extremity function, mobility, and independence in this vulnerable population, thereby balancing aggressive oncologic treatment with enhanced long-term quality of life and reduced treatment-related morbidity.

## Simple Summary

Breast cancer is the most common cancer in women, and when it spreads to the lymph nodes in the armpit, surgeons often need to remove them—a procedure called axillary lymph node dissection, or ALND. This surgery is effective against cancer, but many patients develop long-term arm swelling known as lymphedema, which causes chronic discomfort, limits arm movement, and makes daily tasks genuinely difficult—especially for older women managing other health conditions at the same time. Axillary reverse mapping (ARM) uses a fluorescent dye injected into the arm before surgery to light up the arm’s own lymphatic channels under a special camera, allowing the surgeon to protect them while still removing the cancer-related nodes. In this study, 72 older women who underwent ALND with ARM were compared to 66 who had standard ALND. Those who had ARM were far less likely to develop significant arm swelling—18.1% versus 60.6%—while cancer recurrence rates were similar in both groups, confirming that protecting these vessels did not compromise cancer treatment. ARM appears to be a safe, practical addition to breast cancer surgery that can meaningfully reduce one of its most burdensome long-term complications.

## 1. Introduction

Breast cancer remains the most common malignancy among women worldwide, and axillary lymph node dissection (ALND) continues to play a pivotal role in the surgical management of node-positive disease. However, ALND is associated with substantial postoperative morbidity, most notably breast cancer-related lymphedema (BCRL), which can significantly impair quality of life, cause chronic pain, restrict mobility, and threaten independent living—particularly in older women, where comorbidities and reduced physiological reserve amplify these effects [1,2].

The incidence of BCRL after ALND ranges from 14 to 50% (up to 30–50% with adjuvant regional nodal irradiation), depending on assessment methods, follow-up duration, and risk factors [1,3,4,5,6,7,8]. In contrast, sentinel lymph node biopsy alone carries a much lower risk (5–13%) [1,3,9,10,11]. Axillary reverse mapping (ARM) was developed to address exactly this problem. ICG dye is injected subcutaneously into the ipsilateral hand or forearm before the operation; under near-infrared fluorescence (NIRF) imaging, the arm’s own lymphatic channels become visible and can be distinguished from the breast-draining nodes targeted for removal. The technique has been described for over a decade, though adoption remains uneven—centers without NIRF equipment or experience with the learning curve have been slower to implement it. ARM has emerged as a promising approach, with recent meta-analyses reporting pooled odds ratios of 0.20–0.30 in favor of ARM for reducing BCRL [5,6,7].

The broader surgical context is also shifting. Targeted axillary dissection (TAD)—combining sentinel lymph node biopsy with removal of the clipped biopsy-proven node—has become an established alternative to ALND in patients with limited axillary disease, including after neoadjuvant chemotherapy [12,13,14], and is increasingly used at centers where appropriate staging allows it. For patients who still require full ALND, however, reducing the morbidity of that surgery remains a clinically meaningful goal, and ARM is currently the most developed technique for doing so [15].

Despite these encouraging results, critical gaps remain: most studies have heterogeneous follow-up, variable lymphedema definitions, and limited data in older and frail populations, where treatment-related morbidity has the greatest impact on independence, quality of life, and societal burdens such as caregiver dependency [7,8,16]. Concerns regarding oncologic safety due to potential crossover (reported rates of 12–19%) also persist [17,18].

The present study evaluates the impact of ARM on both the incidence/severity of lymphedema and its implications for quality of life in an older adult cohort undergoing ALND. We hypothesized that ARM would significantly reduce clinically relevant lymphedema (Grades 2–3) and thereby preserve upper-extremity function, mobility, and independence, contributing to enhanced long-term quality of life while maintaining comparable oncologic outcomes.

## 2. Materials and Methods

### 2.1. Study Design and Patient Allocation

This retrospective cohort study included 138 consecutive female patients who underwent ALND for invasive breast cancer between January 2018 and January 2024 at a single tertiary care center. Patients were stratified into two groups: the ARM group (n = 72) and the standard ALND (non-ARM) group (n = 66). The study was approved by the Non-Interventional Clinical Research Ethics Committee (approval number: 2025/04/08/040) and conducted in accordance with the Declaration of Helsinki. Informed consent was waived due to the retrospective design.

Allocation to the ARM group was non-randomized and determined by the operating surgeon’s adoption phase of the technique. ARM was introduced in January 2018 using intraoperative near-infrared fluorescence (NIRF) imaging (Stryker SPY Elite, Stryker, Kalamazoo, MI, USA) with ICG (ICG; Verdye, Diagnostic Green GmbH, Aschheim, Germany). During the initial learning curve phase, patients were allocated to standard ALND if ARM had not yet been routinely implemented. To minimize technical variability and performance bias, the first 30 ARM procedures performed by the surgeon were excluded from the final analysis. This exclusion threshold was based on prior literature demonstrating that ARM identification rates stabilize after approximately 20–50 procedures per surgeon [16,17]. As surgeon proficiency increased, ARM became the standard approach within the same experienced breast surgery team (>10 years of expertise in breast cancer surgery), while adhering to identical postoperative oncologic protocols.

A small number of patients during the ARM period were allocated to standard ALND—primarily in cases where comorbidities made a longer procedure inadvisable, or where the complexity of the combined procedure did not allow for ARM. These patients were included in the non-ARM group. The study period overlaps with the COVID-19 pandemic (2020–2021), which reduced elective surgical volumes temporarily; however, inclusion criteria remained unchanged throughout, and a sensitivity analysis excluding those two years did not alter the primary outcomes.

### 2.2. Inclusion and Exclusion Criteria

Patients were eligible for inclusion if they met the following criteria: (1) histologically confirmed invasive breast cancer; (2) underwent ALND (either as a primary procedure or following SLNB); (3) availability of complete baseline and postoperative assessment data, including serial arm volume measurements or bioimpedance spectroscopy (BIS); and (4) a minimum documented follow-up of 24 months.

Exclusion criteria were defined as: (1) bilateral breast cancer; (2) history of prior axillary surgery or regional nodal radiotherapy; (3) pre-existing lymphedema from secondary causes; and (4) incomplete clinical records or loss to follow-up before the 24-month threshold. To ensure technical proficiency, the first 30 cases were excluded as part of the learning curve. Additionally, patients with incomplete follow-up or those not meeting the inclusion criteria were excluded from the analysis.

### 2.3. Surgical Technique

In the ARM group, reverse mapping was performed using indocyanine green (ICG; 0.5–1 mL injected subcutaneously into the dorsum of the ipsilateral hand or forearm), visualized via a NIRF imaging system. Fluorescent lymphatics and nodes draining the upper extremity were identified and preserved whenever oncologically feasible. Simultaneously, standard Level I–II ALND was performed to resect breast-draining nodes. In the non-ARM group, standard Level I–II ALND was performed without any lymphatic mapping. All procedures adhered to National Comprehensive Cancer Network (NCCN) guidelines [18] and were conducted by experienced breast surgeons.

The addition of ARM added approximately 8–15 min to operative time. This reflects the waiting period after ICG injection for the dye to reach the axillary lymphatics, which typically occurs within 8–15 min of subcutaneous injection [19,20]. Unlike some other technical additions, this time increment is not learning curve-dependent—it is an inherent property of lymphatic transit time and remains consistent regardless of surgical experience.

### 2.4. Lymphedema Assessment

Lymphedema evaluation was performed preoperatively and postoperatively at 3, 6, 12, 24, 36, 48, and 60 months. To ensure objectivity and minimize observer bias, all assessments were performed by three breast care nurses with specific training in lymphedema evaluation, each blinded to group allocation throughout the follow-up period. Arm volumes were calculated using serial circumference measurements (taken at 10 cm intervals from the wrist to the axilla) applied to the Frustum formula. Where available, BIS was used to supplement physical measurements using a validated bioimpedance device.

Lymphedema severity was graded from 0 to 3 according to criteria adapted from the International Society of Lymphology (ISL) criteria [21]:

- *Grade 0:* No lymphedema (normal arm, no measurable volume increase compared to the contralateral side, and no BIS changes).
- *Grade 1:* Subclinical/latent lymphedema (no visible swelling; mild increase in lymphatic load or BIS alterations; corresponding to ISL Stage 0).
- *Grade 2:* Mild lymphedema (clinically visible swelling with <20% volume difference compared to the contralateral arm; corresponding to ISL Stage I).
- *Grade 3:* Moderate lymphedema (≥20% volume difference and associated functional impairment; corresponding to ISL Stage II).

For statistical analysis, “clinically relevant lymphedema” was defined as Grades 2–3. Grade 1 represented subclinical/latent changes. Oncologic outcomes, including locoregional recurrence and distant metastasis, were monitored throughout the follow-up period.

### 2.5. Data Collection

Demographic (age, BMI), clinicopathologic (tumor size, histological grade, molecular subtype, Ki-67 index, lymphovascular invasion [LVI], and TNM stage), and treatment-related data (neoadjuvant therapy, type of surgery, and total/metastatic nodes removed) were retrieved from the institution’s electronic medical records. Lymphedema assessment data, including serial arm circumference measurements and BIS values, were systematically collected. Arm volumes were subsequently calculated using the Frustum formula, and lymphedema was staged according to the ISL criteria. Follow-up data regarding oncologic outcomes, such as locoregional recurrence and distant metastasis, were also recorded.

### 2.6. Statistical Analysis

Statistical analyses were performed using SPSS version 27.0 (IBM Corp., Armonk, NY, USA). Before selecting between parametric and non-parametric tests, we assessed normality for all continuous variables using the Shapiro–Wilk test. Most variables, including age, follow-up duration, and node counts, showed non-normal distributions, which supported the use of the Mann–Whitney U test for group comparisons. Distribution plots for all continuous variables are provided as Supplementary Figures S1–S4. Continuous variables were compared using the Mann–Whitney U test, and categorical variables using the chi-square or Fisher’s exact test, as appropriate. Kaplan–Meier analysis with log-rank testing was used for time-to-event outcomes. Multivariable Cox regression was performed to identify independent predictors of clinically significant lymphedema. Hazard ratios (HRs) with 95% confidence intervals (CIs) were reported. A *p*-value < 0.05 was considered statistically significant. 

## 3. Results

### 3.1. Study Population

Between January 2018 and January 2024, 138 consecutive female patients who underwent ALND for invasive breast cancer were included in this retrospective cohort study. Patients were categorized into two groups according to whether ARM was performed during ALND (ARM group, n = 72) or not (standard ALND, n = 66). Twenty-two patients were excluded due to insufficient follow-up or incomplete medical records. All included patients had a minimum follow-up duration of 24 months.

### 3.2. Baseline Characteristics

Baseline demographic and clinicopathologic features were comparable between groups (Table 1). Mean age was 72.0 ± 4.3 years in the ARM group and 73.0 ± 4.8 years in the non-ARM group (*p* = 0.712). Stage III disease was the most common presentation in both cohorts (69.4% vs. 63.7%, *p* = 0.512). Molecular subtype distribution was similar (*p* = 0.906), with luminal A being the most common. Neoadjuvant therapy rates were equivalent (70.8% vs. 72.7%, *p* = 0.80). The mean number of total lymph nodes retrieved was comparable (16.4 ± 4.2 vs. 15.8 ± 3.8, *p* = 0.54), indicating that the performance of ARM did not reduce the extent of axillary dissection.

### 3.3. Lymphedema Incidence and Severity

**Figure 1:**
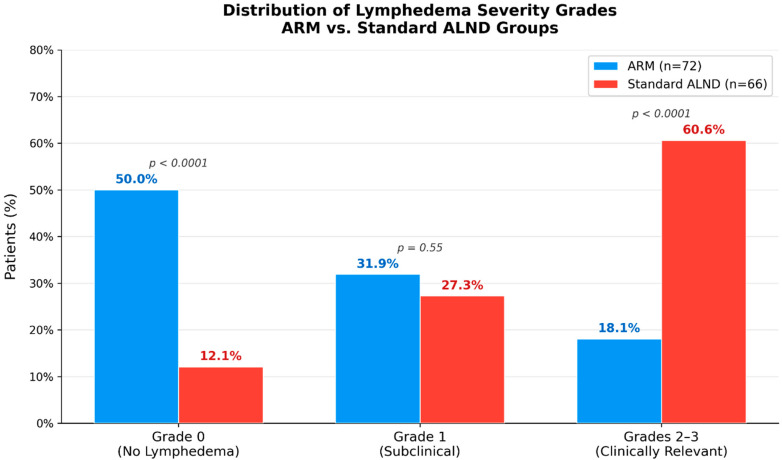
Distribution of lymphedema severity grades in the ARM and non-ARM groups. Severity was graded as follows: Grade 0 (no lymphedema; completely normal arm), Grade 1 (subclinical/latent; no visible swelling but mild lymphatic load increase or BIS changes), Grade 2 (mild clinical), and Grade 3 (moderate/severe clinical). The ARM group showed a significantly higher rate of Grade 0 (50.0% vs. 12.1%, *p* < 0.0001) and lower rate of Grades 2–3 (18.1% vs. 60.6%, *p* < 0.0001), while Grade 1 rates were comparable (31.9% vs. 27.3%, *p* = 0.55).

The incidence of clinically relevant BCRL—the form most strongly associated with impaired quality of life—was dramatically lower in the ARM group (18.1% vs. 60.6%, *p* < 0.0001). Subclinical changes were similar between groups (31.9% vs. 27.3%, *p* = 0.55). The overall incidence of any lymphedema (Score > 0) was also significantly reduced in the ARM group (50.0% vs. 87.9%, *p* < 0.0001). The severity distribution is detailed in Figure 1 and Table 2:

- Grade 0: 50.0% vs. 12.1% (*p* < 0.0001);
- Grade 1: 31.9% vs. 27.3% (*p* = 0.55);
- Grades 2–3: 18.1% vs. 60.6% (*p* < 0.0001).

**Table 2:** Distribution of lymphedema severity.

Grade	ARM (n = 72), n (%)	Non-ARM (n = 66), n (%)	*p*-Value
0 (None)	36 (50.0)	8 (12.1)	<0.0001
1 (Subclinical)	23 (31.9)	18 (27.3)	0.55
2–3 (Clinically relevant)	13 (18.1)	40 (60.6)	<0.0001

Footnote: Values are presented as n (%). Categorical variables were compared using the chi-square or Fisher’s exact test as appropriate. Clinically relevant lymphedema was defined as Grades 2–3 (corresponding to ISL Stages I–II). Statistical significance was set at *p* < 0.05.

**Figure 2:**
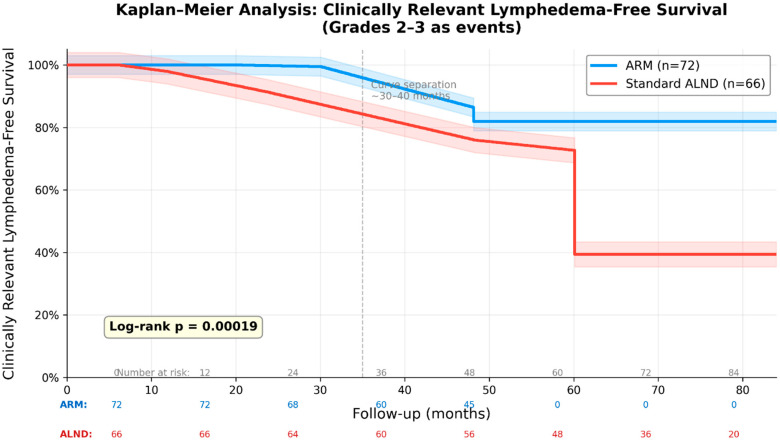
Kaplan–Meier curves for clinically relevant lymphedema-free survival (Grades 2–3 as events) in the ARM and non-ARM groups. The ARM group demonstrated significantly superior lymphedema-free survival (log-rank *p* = 0.00019). Curves begin to separate noticeably after 30–40 months of follow-up, with the benefit persisting throughout the observation period.

Kaplan–Meier analysis demonstrated markedly superior clinically relevant lymphedema-free survival in the ARM group (log-rank *p* = 0.00019; Figure 2), with clear curve separation emerging after 30–40 months. This delayed divergence highlights ARM’s role in preventing late-onset, progressive lymphedema in older patients. When subclinical cases were included, no significant difference was observed (*p* = 0.63).

When the analysis was stratified by ALND type, the pattern was consistent. In the primary ALND group (ARM n = 52, non-ARM n = 45), clinically relevant lymphedema occurred in 11.5% of ARM patients versus 71.1% of non-ARM patients (*p* < 0.0001). In the completion ALND group—patients who had a prior sentinel node biopsy followed by completion dissection (ARM n = 20, non-ARM n = 21)—rates were 25.0% versus 52.4% (*p* = 0.140). The SLNB rates were comparable between groups (27.8% vs. 31.8%, *p* = 0.740), confirming that the subgroups were balanced. The smaller completion ALND subgroup likely lacked sufficient power to reach significance despite a clinically meaningful difference in the same direction.

### 3.4. Oncologic Outcomes

Recurrence rates were comparable between groups (8.3% in ARM vs. 10.6% in non-ARM, *p* = 0.776). No significant differences were found in locoregional recurrence or distant metastasis rates, supporting the oncologic safety of ARM in this cohort.

### 3.5. Multivariable Analysis

Multivariable Cox regression analysis adjusting for age, BMI, neoadjuvant therapy, nodal status, lymphovascular invasion, tumor grade, and molecular subtype demonstrated that ARM was independently associated with a lower risk of clinically relevant lymphedema (adjusted HR 0.22, 95% CI 0.11–0.44, *p* < 0.0001). ARM status was not associated with subclinical changes (*p* = 0.52).

## 4. Discussion

The present study demonstrates that ARM is associated with a dramatic reduction in clinically relevant lymphedema following ALND, without evidence of compromised oncologic safety. By differentiating subclinical volume changes from overt clinical disease, our analysis provides insight into the stage-specific protective effect of ARM on lymphatic morbidity.

Some lymphedema still occurred in the ARM group, and it is worth being specific about why. Crossover nodes—where arm-draining and breast-draining lymphatics anatomically converge, reported in 12–19% of cases in published series [16,17,18]—were identified in 9.7% of our cohort and could not be preserved safely on oncologic grounds. Beyond crossover, smaller collateral channels can be inadvertently damaged during dissection even when the ARM node itself is protected. All patients received adjuvant radiotherapy according to institutional protocol, which routinely includes the chest wall or residual breast, axilla, and supraclavicular region as standard fields; variations in internal mammary irradiation were applied based on individual risk stratification within institutional guidelines rather than selectively [20,22], and therefore did not introduce differential bias between groups. The 18.1% figure likely represents something close to the current floor for this technique, not a failure of the approach [5,6,17].

Clinically relevant lymphedema is the form most strongly associated with impaired quality of life, reduced upper extremity mobility, loss of independence, chronic pain, and psychological burden in older patients. By decreasing its incidence from 60.6% to 18.1% (*p* < 0.0001), ARM offers a meaningful strategy to prevent the “prolonged misery” described by Ivor Lewis in the context of aggressive surgical treatments. The delayed curve separation in Kaplan–Meier analysis after 30–40 months (log-rank *p* = 0.00019) further highlights ARM’s particular value in safeguarding long-term quality of life rather than merely mitigating short-term postoperative changes.

Our primary finding is consistent with previously published meta-analyses reporting odds ratios between 0.20 and 0.30 in favor of ARM for reducing BCRL [22]. Subclinical changes were not significantly different between groups (31.9% vs. 27.3%, *p* = 0.55), suggesting that ARM may not fully prevent early lymphatic disturbance but may attenuate progression toward clinically manifest lymphedema [5,6,17]. The delayed separation of survival curves further indicates that the principal benefit of ARM may lie in preventing chronic, late-onset clinically relevant disease.

Oncologic safety remains a critical consideration for ARM adoption. In this cohort, recurrence rates were comparable (8.3% vs. 10.6%, *p* = 0.776), and the number of retrieved lymph nodes did not differ significantly (16.4 ± 4.2 vs. 15.8 ± 3.8, *p* = 0.54). Multivariable analysis confirmed that ARM was not an independent predictor of recurrence, indicating that preservation of arm-draining lymphatics did not compromise axillary clearance or short- to mid-term oncologic outcomes [23,24].

The study population consisted predominantly of older patients, a demographic frequently underrepresented in prior ARM investigations. Given the higher baseline risk of functional decline and comorbidity in this age group, strategies that reduce treatment-related morbidity may be especially important for maintaining independence and reducing broader societal burdens, including caregiver dependency and healthcare utilization [25,26]. The age distribution reflects where our center sits: a tertiary referral hospital with an older catchment population, where ALND indications have progressively concentrated in older patients with heavier disease as younger patients with limited nodal burden have been redirected toward less extensive axillary surgery [12,13,27].

Strengths of the study include blinded assessments by trained nurses, standardized limb volume calculations using the Frustum formula supplemented by BIS, exclusion of the initial 30 cases to account for the surgical learning curve, and long-term follow-up. These methodological features enhance internal validity and reduce measurement and performance bias.

Several limitations warrant consideration. The retrospective design and non-randomized allocation introduce potential selection bias, although multivariable adjustment and comparable baseline characteristics mitigate this concern. The single-center setting and focus on an older population may limit generalizability to younger or multicenter cohorts. Lymphedema severity was assessed objectively using standardized circumference measurements and BIS at predefined time points throughout follow-up. Formal patient-reported outcome measures were not collected as part of this study’s protocol—subjective symptom burden, daily activity limitation, and psychological impact were not directly captured. The ISL grading system we used reflects clinically measurable disease severity, which correlates well with functional impairment in the literature, but does not substitute for what patients themselves report. This is a limitation we acknowledge, and incorporating validated instruments such as the LYMPH-Q [28] in future prospective work would strengthen the patient-centered evidence base for ARM.

In the context of an aging breast cancer population, these findings have direct clinical and societal relevance. Preservation of arm function translates into maintained independence, reduced caregiver burden, and lower long-term healthcare costs—key considerations in modern value-based surgical oncology. Centers considering adopting ARM should expect a learning curve of roughly 20–50 cases before identification rates stabilize [17,18]—we excluded our first 30 for this reason. Equipment costs are real: a near-infrared imaging system and ICG are not trivial additions. That said, chronic lymphedema is expensive to manage, with annual costs per patient running into thousands of dollars [29,30] when compression garments, physiotherapy, and outpatient visits are factored in. In our setting ICG is covered by the national health system, which simplifies the calculation considerably. The broader trend toward TAD should also be acknowledged—as fewer patients require ALND overall, those who do need it are precisely the group where preventing lymphedema matters most [12,13].

## 5. Conclusions

ARM using ICG is a technically feasible and oncologically safe adjunctive approach that significantly reduces the risk of clinically relevant lymphedema following ALND. While ARM does not prevent early subclinical lymphatic alterations, it may effectively limit progression to clinically manifest disease, as evidenced by the substantial reduction in severe cases.

In this predominantly older adult population, where treatment-related morbidity disproportionately affects mobility, independence, and long-term quality of life, ARM offers a practical, patient-centered strategy to mitigate the debilitating impact of breast cancer-related lymphedema without compromising axillary clearance or short- to mid-term oncologic outcomes.

These findings support the consideration of ARM in appropriately selected older adults, balancing aggressive oncologic surgery with preservation of functional independence and enhanced quality of life, pending confirmation in larger prospective studies with extended follow-up and direct patient-reported outcome measures.

## Figures and Tables

**Table 1:** Baseline demographic, clinicopathologic, and treatment characteristics.

Variable	ARM Group (n = 72)	Non-ARM Group (n = 66)	Total (n = 138)	*p*-Value
**Age, years (mean ± SD)**	72.0 ± 4.3	73.0 ± 4.8	72.5 ± 4.5	0.712
**Follow-up duration, months (mean ± SD)**	46.0 ± 8.5	47.0 ± 9.1	46.5 ± 8.8	0.681
**Molecular Subtype, n (%)**				0.906
Luminal A	26 (37.1)	28 (41.2)	54 (39.1)	
Luminal B	22 (31.4)	19 (27.9)	41 (29.7)	
HER2-positive	11 (15.7)	12 (17.6)	23 (16.7)	
Triple-negative (TNBC)	11 (15.7)	9 (13.2)	20 (14.5)	
**Clinical Stage, n (%)**				0.512
Stage I	2 (2.8)	2 (3.0)	4 (2.9)	
Stage II	20 (27.8)	22 (33.3)	42 (30.4)	
Stage III	50 (69.4)	42 (63.7)	92 (66.7)	
**Neoadjuvant Therapy, n (%)** **Yes/No**	51/21 (70.8/29.2)	48/18 (72.7/27.3)	99/39 (71.7/28.3)	0.80
**Type of Surgery, n (%)**				0.09 †
Mastectomy	36 (50.0)	43 (65.2)	79 (57.2)	
Breast-conserving surgery	21 (29.2)	15 (22.7)	36 (26.1)	
Other (combined/complex procedures)	15 (20.8)	8 (12.1)	23 (16.7)	
**Recurrence, n (%)**	6 (8.3)	7 (10.6)	13 (10.1)	0.776
**Any lymphedema (Score > 0), n (%)**	36 (50.0)	58 (87.9)	94 (68.1)	<0.0001

Footnote: Values are presented as mean ± standard deviation (SD) or n (%). Continuous variables were compared using the Mann–Whitney U test. Categorical variables were compared using the chi-square or Fisher’s exact test as appropriate. † Overall comparison across surgical categories.

## Data Availability

The data supporting the findings of this study are available from the corresponding author upon reasonable request. Public sharing is not possible due to patient privacy restrictions and institutional ethical guidelines governing retrospective clinical data.

## Supplementary Materials

The following supporting information can be downloaded at: https://www.mdpi.com/article/10.3390/curroncol33040212/s1, Figure S1: Normality assessment—Age (years). Figure S2: Normality assessment—Follow-up duration (months). Figure S3: Normality assessment—Total lymph nodes retrieved. Figure S4: Normality assessment—Total positive lymph nodes. Shapiro–Wilk test results and Q-Q plots are presented for each variable.
