# Supplementary figures and images for: Axillary Reverse Mapping Improves Quality of Life by Significantly Reducing Clinically Relevant Lymphedema After Axillary Lymph Node Dissection in Older Women with Breast Cancer
Source: Curr Oncol. 2026 Apr 10;33(4):212. doi: 10.3390/curroncol33040212 (PMC13114941; doi:10.3390/curroncol33040212)

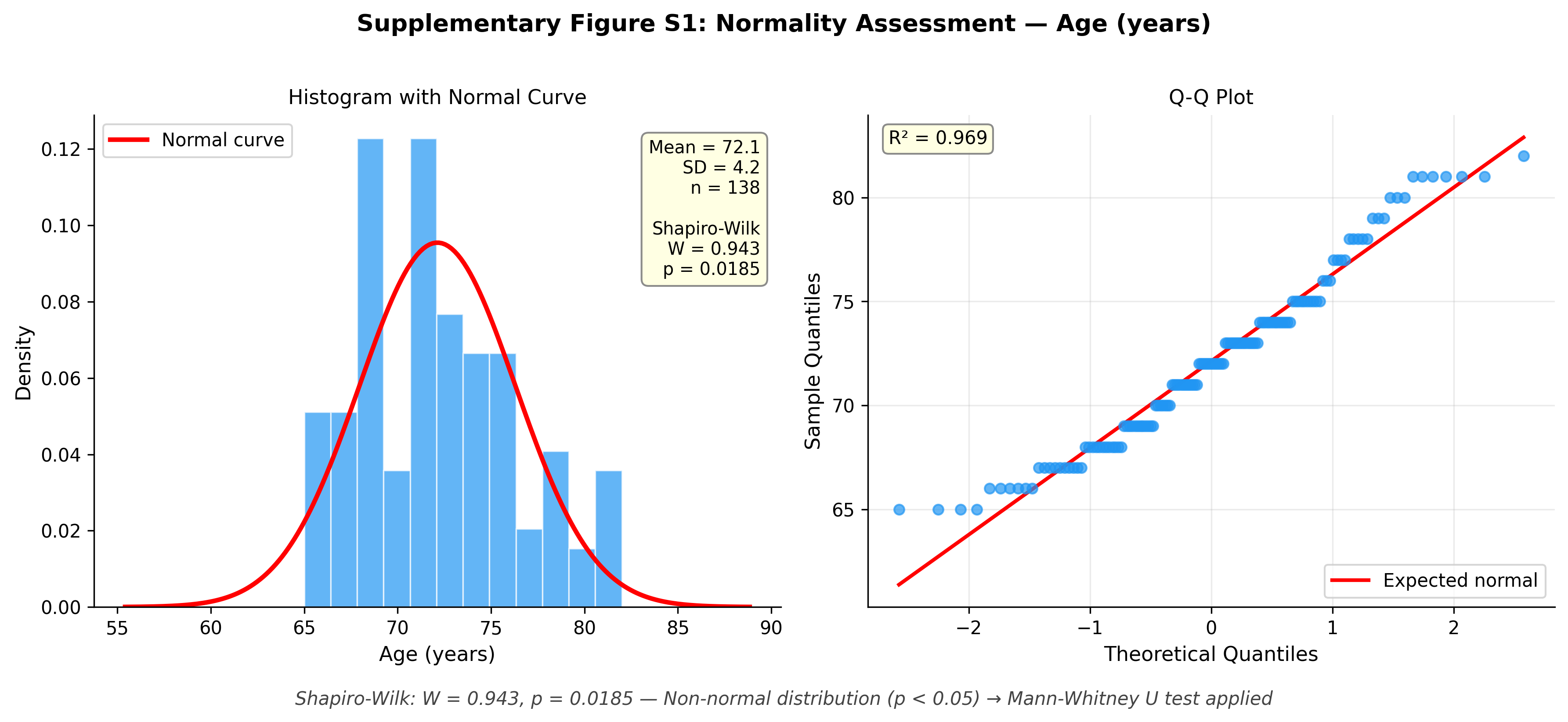

Supplement: Supplementary file 1 [file curroncol-33-00212-s001.zip › Supplementary_Figure_S1_normality.png]

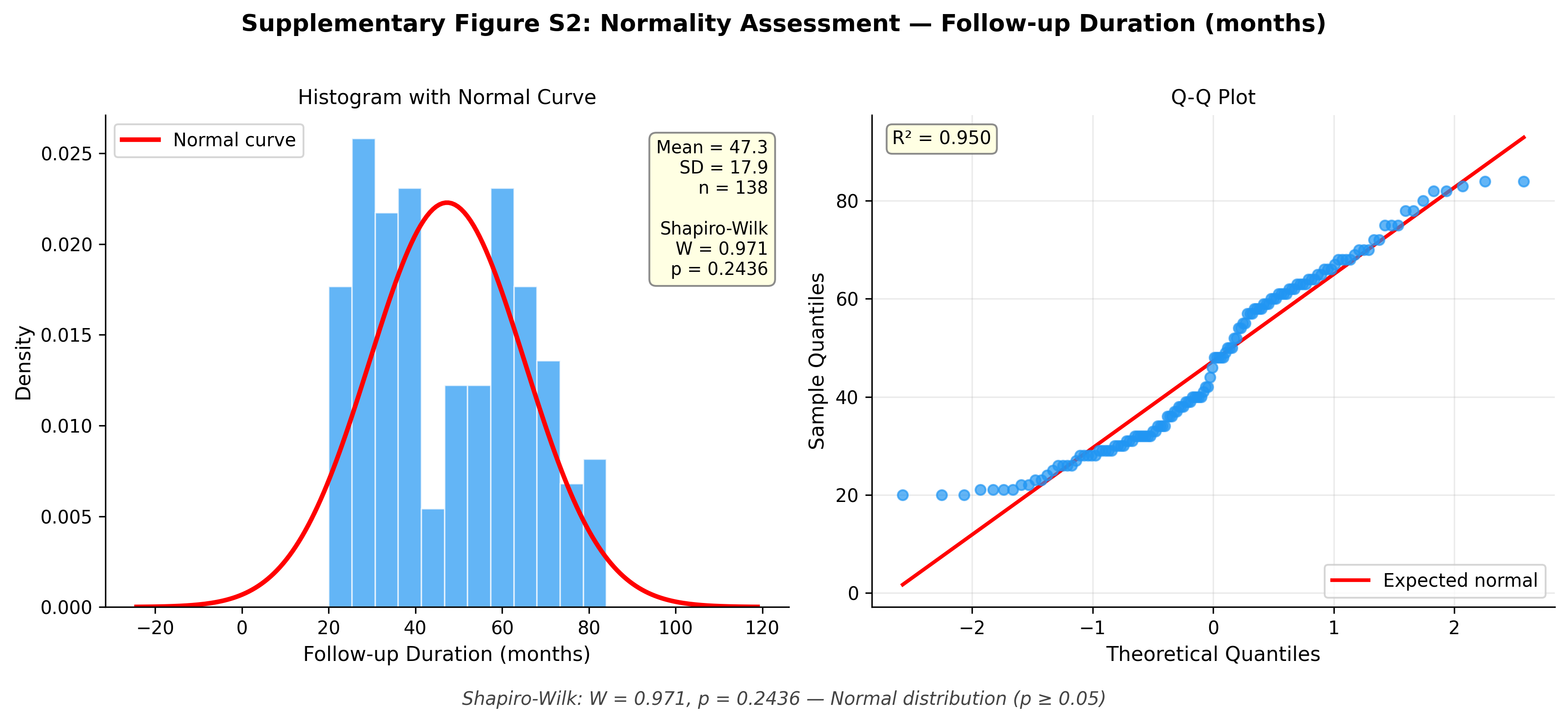

Supplement: Supplementary file 1 [file curroncol-33-00212-s001.zip › Supplementary_Figure_S2_normality.png]

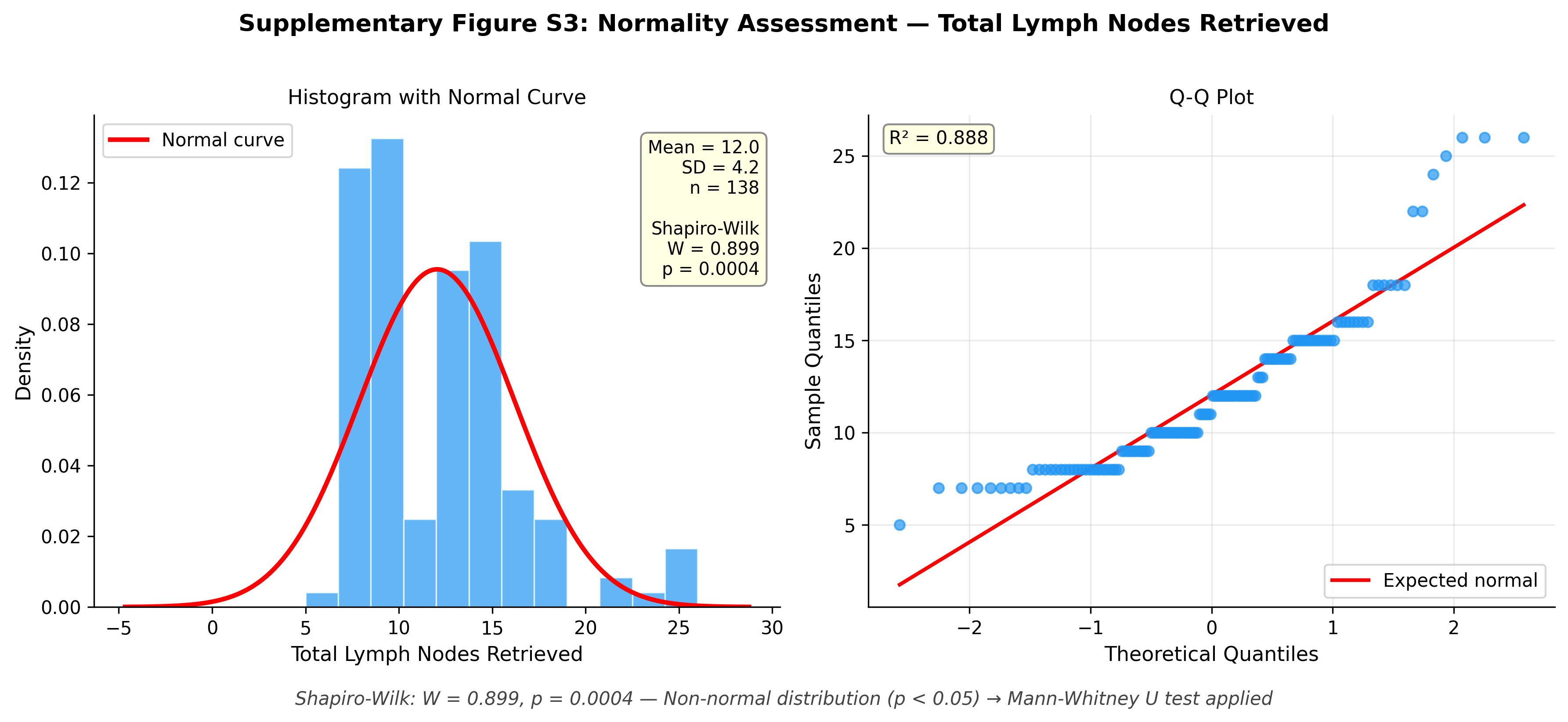

Supplement: Supplementary file 1 [file curroncol-33-00212-s001.zip › Supplementary_Figure_S3_normality.png]

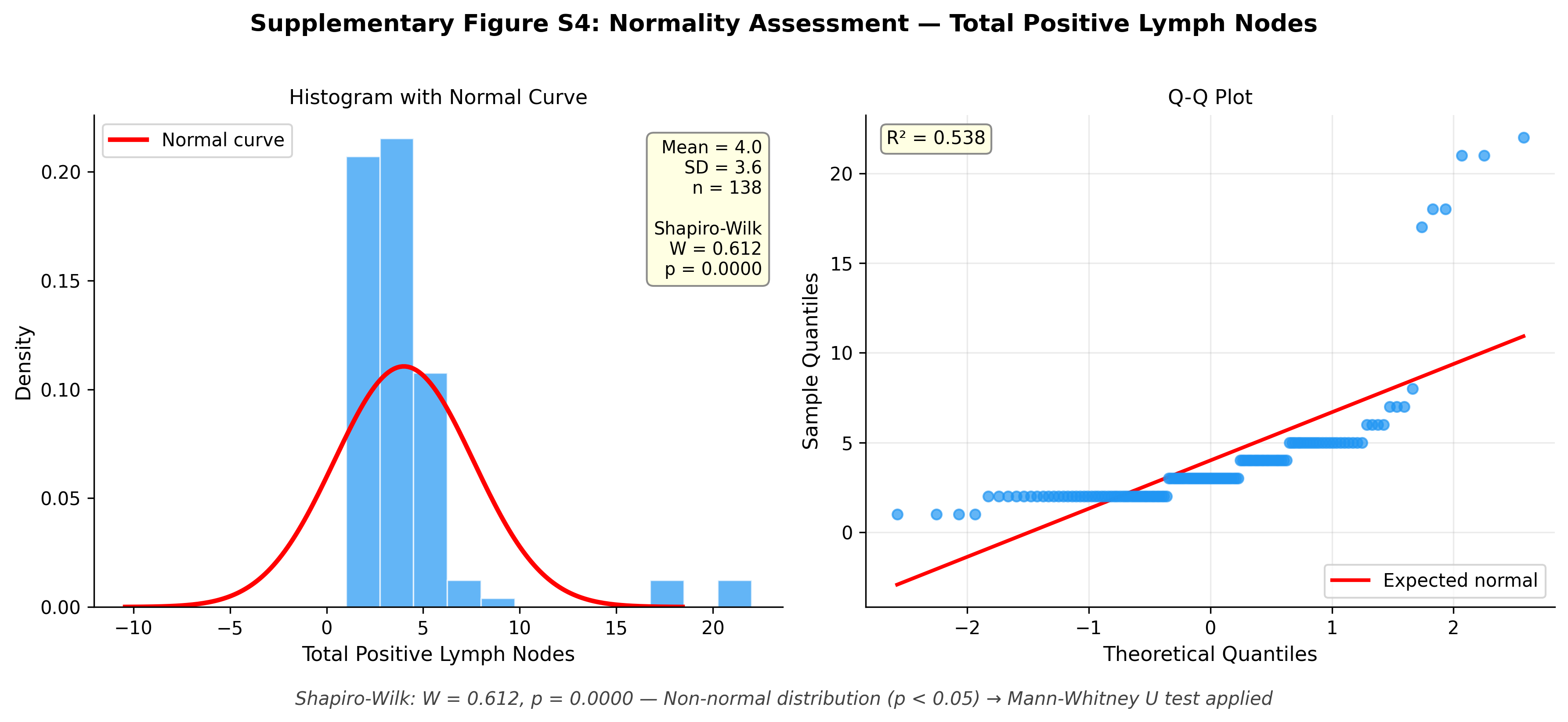

Supplement: Supplementary file 1 [file curroncol-33-00212-s001.zip › Supplementary_Figure_S4_normality.png]
